# Mr. Wadd's Mems. Maxims, and Memoirs

**Published:** 1827-10

**Authors:** 


### *Mems. Maxims, and Memoirs.* By William Wadd, Esq. F. L. S. Surgeon Extraordinary to the King, &c. &c. &c.

Quidquid agunt *Medici,*
Nostri est farrago libelli.

In our 13th Number we introduced two *novel* productions from two Collegiate *fellows* of this Metropolis, viz. "Nugæ Canoræ," and the "Gold-headed Cane," for the edification and amusement of our readers, many of whom, from the crowded state of the profession, have doubtless abundance of time on their hands, for light recreations of this kind. The present volume is from the pen of Mr. Wadd, (to whom Fame attributes the Nugæ Canoræ,) and contains a great variety of Mems. Maxims, and Memoirs, as the alliterated title quaintly informs us, collected, with more labour than many of them are worth, from the musty records where they had long slept in well-merited oblivion. Take two or three at hap-hazard:—"1746, July 3, the Members of the Corporation of Surgeons invited to dine with the Court of Assistants."—"1712, Dr. Charles Goodall, President of the College, died."—"1721, Controversy on the Plague."—"1556, Snuff first taken by Catherine de Medici, and called *herbe à la Reine.*" These are pretty good samples of the Memoranda, occupying 28 pages of the work. Mr. Wadd might certainly have purged this part of his book of a great mass of "perilous stuff," which must lie as a dead weight on the production. Before we quit this department, however, we shall quote two or three other memoranda, as matters of curiosity. Thus, in 1680, we find the "Marquis of Dorchester, made a Fellow of the College of Physicians."—in 1750, "died, his grace, Charles, Duke of Richmond, Fellow of the Royal College of Physicians"—and the year before, we find John Duke of Montagu, to have departed this life, although a Fellow of the College of Physicians. In that same year, with such high personages in the fellowship, we do not wonder to find it placed on record by Mr. Wadd, that "Dr. Addington refuses to consult with a Licentiate."

The following piece of information follows as a natural consequence. "1767, Sept. 30, at the Anniversary of the College of Physicians, the Licentiates demanded admittance, which was not complied with. A smith was offered ten guineas, and an indemnification of 300 pounds, to force the gates, which he refused." They will probably take a wiser way of proceeding in the present day—when reason and common-sense may effect what the smith's hammers and crow-bars would fail to do.

### MEMORABILIA.

Under the head of "*fees*," we have some curious facts and shrewd comments on them. It is but too well known how happy is the patient to see his doctor when he is in great suffering, or supposes himself to be in great danger. It is then, "My dearest friend," and the like. When recovering a little, it is *my good Dr., or Mr. So and So;* but the pain gone, and the danger over—it is simply good morning Dr., or Mr., without any endearing expression. Every day's experience, says Mr. Wadd, shews that "accipe dum dolet," should be the medical man's motto. He farther remarks, and very justly, that the advancement of surgical science is a benefit conferred on society, at the expense of the scientific practitioner, since, in proportion as the mode of cure is, "celeriter et tuto," so is the remuneration diminished. The operation for hydrocele exemplifies this: compare the simplicity, safety, and celerity of the present, with the bustle and bloody brutality of the old system! The business of six weeks is reduced to as many days. What is the consequence as to the *honorarium?* Does the patient increase the fee? Not a bit of it! Here is little or no *work done*—no trouble to the doctor—no pain to the patient—therefore little to pay for. But let an ignorant blunderer be six weeks remedying the effects of his own mischief—or let Nature triumph over bad practice, and we shall see what gratulations and gratuities flow to the great Apollo!

The *celeriter*, however, in the healing art, has not outstripped the improvements of the age in many things, whatever may be said as to the *tuto*. It took our forefathers a whole day to go to Brighton or Margate—which we can now do in six hours—with the trifling risk of a blow up by steam, or a few fractures by the upsetting of a *celerity* safety-coach!

The modes of medical remuneration are various in different countries. The Chinese mode of stopping the court physician's salary while the emperor is ill, forms no simple trait in the character of that crafty people. We are informed by Southey, in his History of the Peninsular War, that Mr. T. an English surgeon, was rewarded for curing a dangerous wound in the abdomen of a court domestic, by having his picture hung up in Lapa Church, surmounted by that of the Virgin Mary, "who had enabled him to perform the cure." This was rather a disappointment, we should suppose, to the English surgeon, but he fared better than the two physicians of Austrogilda, queen of Burgundy, who were slain and buried with her majesty, by her own dying wish? These were probably the only two medical gentlemen who were ever priviledged to lie in the tombs of kings and queens. On the other hand, on the death of Pope Adrian, the door of the physician's house was ornamented with a garland, bearing the inscription—"*To the deliverer of his country.*"

The following extract respecting the first founders of the College of Physicians, will not be uninteresting at the present day.

"It is singular that the college should be founded by persons who took their degrees of medicine out of the kingdom, and one of whom (Dr. Ferdinand de Victoria) was a foreigner.

"Dr. Chambre graduated at Padua; Dr. Linacre graduated in Italy; Dr. Caius graduated at Bononia; Dr. W. Harvey at Padua; Dr. Hamey at Leyden; Sir John Micklethwaite at Padua.

"Caius, Harvey, and Sir John Micklethwaite were afterwards *incorporated* Doctors of English Universities, a circumstance particularly noticed by Wood.—*Fasti*, vol. i. p. 8." 63.

Under the date of 1572, we find that it was debated at the Mansion-house, before the Lord Mayor, the Bishop of London, the Master of the Rolls and others, "whether a surgeon could lawfully prescribe internal remedies," when it was decided that he could not—"no, not even in *morbo gallico*." "A surgeon was afterwards mulcted for so prescribing." In the 32d of Henry VIII. the College of Physicians inserted a clause, stating that physic comprehended surgery, and therefore "Doctors might practise surgery." In 1595, the College intimated by letter to the Surgeons' Company, their intention to proceed against all who should offend in this matter. In 1602, Chief Justice Popham gave a judgment "which produced a very flattering letter of thanks from the College." How greatly are things changed since that time! Yet some of the laws or regulations kept up to this day, are equally absurd, and far more unjust than many that have become obsolete from the alteration in times and circumstances.

From this long division of our author's work, occupying more than 160 pages, we are sorry to say that we can find little or nothing which we dare to record on our pages. The term MEMORABILIA is decidedly a misnomer—it ought to have been "OBLIVISCENDA," as containing a "rudis indigestaque moles" of things that cannot be turned to any useful account, and which can only gratify curiosity, or provoke smiles at the follies, eccentricities, or knavery, of some of our professional forefathers!

The third and last division of the work, entitled "MEMOIRS," is more interesting and useful, as consisting of short biographical sketches of men who have "strutted their hour" on the medical stage to sink and make way for others. Of some of these characters and portraits we shall take some short notice.

1. *Sir Theodore Mayerne.* This great chemical doctor having been driven from France, was received in England, and incorporated Doctor of Medicine at Oxford, anno 1606. He soon became chief physician to King James I. and appears to have dabbled in many branches of the profession, especially midwifery and *cookery*. He was also a great pharmacologist, and a bit of a horse-doctor (saving the presence of the Universities) for he has left us the case, and the cure of the Queen's black horse, who was affected with epilepsy. The case is reported, of course, in the Latin language, and begins thus:—"Equus est novem annorum," &c. and he states that the horse "curatus fuit." In short, this Fellow of the College, and physician to four kings, was as arrant a quack as ever wrote his name on wall or hand bill. As to ignorance, superstition, and credulity, his powder for the gout, containing *raspings of the human skull*—his remedy for hypochondriasis, consisting of *adders, bats, sucking whelps, earth-worms, hog's grease, marrow of a stag, &c.* are sufficient specimens. These precious formulæ were introduced into the official pharmacopœia by this celebrated royal physician, on whose tomb-stone was inscribed—" *alter Hippocrates.*"

2. *Sir William Paddye.* The principal incident in the life of this eminent fellow of the College is as follows :—The College was charged with arms, in the reign of King James, but being then very peaceably inclined, Sir William was appointed to plead the privilege of the College before the Lord Mayor and a full court of Aldermen. In this he was not only successful, but obtained the issue of a precept " *to commit all doctors not licensed members of the College.*" Like the devil in a storm, Sir William appears to have been double diligent in his day, and his services might be useful at the present time, when there are so many *licentious doctors* on the town.

3. *Dr. Harvey.* This most erudite physician and Fellow of the College, managed to live a peaceable life in the midst of the civil wars. He hated the hypocritical cant of that day; but thinking it best to go with the stream, he had some favourite classics done up as prayer-books, by means of which he contrived to amuse a dull hour during the ranting of some cobler or tinker. Among other liberal and patriotic donations, he left to the College " an unicorn's horn set in gold," which the College afterwards presented to King Charles, doubtless in gratitude for that merry monarch's kind letter to the College, forbidding the admittance into the fellowship of all ultra-marine graduates—a measure, by the bye, which reflected not a little on the original founders of the College, including Linacre, who were all foreign graduates !

4. *Sydenham.* Of this Father of English Physic little is said by Mr. Wadd, except offering an apology for his leaving London during the plague. It is not every one who is blessed with courage to meet this enemy voluntarily. Many reasons have been surmised for Sydenham's flight—and probably they are all wrong. Might not the cause be, a conviction in his own mind that his stay would do no good to the sick, and might endanger his own life.

> " He who fights and runs away,
> " May live to fight another day."

Dr. Hodges who did stay in London, lost all his practice, and was rewarded with the glory of being the supposed depository of that contagion to which he had been so much exposed. Sydenham came back when the danger was over, pure as unsunned snow—and monopolized an immense run of practice. Ainsi va le monde!

5. *Radcliffe.* Of this medical hero we recorded some traits while noticing the Gold-headed Cane. Mr. Wadd, says—" it is greatly to be lamented that the professional sagacity and decisive character of such a man should live only in tradition." If it be true that " he had as great a contempt for physic as he had for physicians, and that it was his avowed opinion that the whole mystery of the art might be written on half a sheet of paper," we can only regret that the Doctor did not leave this said half-sheet of paper behind him, as a proof and memorial of his ignorance and presumption—two qualities that have a surprising predilection for travelling in company. We do not at all wonder that, before the end of his career, he found himself incapable of curing diseases —for he evidently knew nothing about the nature of them. His prediction (if it deserve that name) of the Duke of Beaufort's death (vide page 40 of No. 13) was any thing but " the result of great talent:"—on the contrary, if it ever was made, and proved to be so correct as is stated, (which we entirely disbelieve) it was a piece of the most barefaced presumption on record. It is evident that he was a free liver of the first water, and died a penitent, afraid of punishment for his convivial excesses. He wrote a long letter to an old pot-companion, Lord Denbigh, in the hope of persuading his lordship to abandon his wicked life ere it was too late. It does not appear that the Doctor himself relinquished the pleasures of the table and the orgies of Bacchus, till age had incapacitated him for their enjoyment. It is at this period that many pious resolutions are made, and many praise-worthy reforms put in practice!

6. *Sir Hans Sloane.* This illustrious character was a contemporary of Dr. Radcliffe, but they were never on good terms—at which we do not much wonder. On his arrival in London, he waited on Sydenham, with a letter of recommendation, setting forth his classical, botanical, and anatomical acquirements. Sydenham, as may be supposed, did not set a very high value on these accomplishments.

" After Sydenham had perused this eulogy, and had eyed the Tyro very attentively; he said, ' all this is mighty fine! but it won't do. Anatomy—botany—nonsense! Sir, I know an old woman in Covent Garden, who understands botany better; and as for anatomy, my butcher can dissect a joint full as well —no, young man, all this is stuff; you must go to the bed-side, it is there you can alone learn disease!' " 231.

Nevertheless Sydenham afterwards patronised Sir Hans, who rose to great celebrity, though not to equal fame with his less learned patron.

Here we must close our short *sketch* of this Medical *Sketch-book*, which contains an amazing mass of " odds and ends," which must, we think, have cost the author more labour than the construction of a work that might have carried down his name to posterity. But every man to his fancy. We doubt not, that the collection of these anecdotes and biographical scraps has afforded Mr. Wadd infinite pleasure and amusement—more perhaps than the perusal will afford to the generality of readers.

